# RT-qPCR Testing of SARS-CoV-2: A Primer

**DOI:** 10.3390/ijms21083004

**Published:** 2020-04-24

**Authors:** Stephen A. Bustin, Tania Nolan

**Affiliations:** 1 Medical Technology Research Centre, Faculty of Health, Education, Medicine and Social Care, Anglia Ruskin University, Chelmsford, Essex CM1 1SQ, UK; 2 Institute of Population Health, Faculty of Medical and Human Sciences, University of Manchester; Manchester M13 9NT, UK; tanianolan@btinternet.com; 3 The Gene Team, Bury St Edmunds, Suffolk IP31 1AA, UK

**Keywords:** COVID-19, SARS, pandemic, reverse transcription, real-time fluorescence PCR

## Abstract

Testing for the presence of coronavirus is an essential diagnostic tool for monitoring and managing the current COVID-19 pandemic. The only reliable test in current use for testing acute infection targets the genome of SARS-CoV-2, and the most widely used method is quantitative fluorescence-based reverse transcription polymerase chain reaction (RT-qPCR). Despite its ubiquity, there is a significant amount of uncertainty about how this test works, potential throughput and reliability. This has resulted in widespread misrepresentation of the problems faced using this test during the current COVID-19 epidemic. This primer provides simple, straightforward and impartial information about RT-qPCR.

## 1. Introduction

There can be little doubt that worldwide governmental and public health organisation responses to the current COVID-19 outbreak have been far from ideal. There have been huge differences in the pursuit of the most appropriate policies for, and effective methods of, testing potential carriers, their contacts, health workers and other emergency service workers. Given that on 9th January 2020 SARS-CoV-2 was definitively identified by the Chinese CDC as the causative agent for COVID-19 pneumonia and that its genomic sequence (GenBank accession number MN908947) was made available on 10th January, it is extraordinary that by the time the earliest documented transmission within the UK appeared on 28th February, no definitive action plan, stockpile of assays and required consumables, RNA extraction robots or high throughput qPCR instruments had been assembled to allow immediate and widespread RT-qPCR testing. Furthermore, the unwillingness to decentralise testing, both with regards to which assays were being used and where the testing was carried out, contributed to a farcical situation, on 11th March, where an increase from 1500 to 10,000 tests per day was considered to be a positive achievement.

The testing situation was further confused by the lack of distinction between PCR-based tests, which detect the viral RNA, and immunoassays, which detect the presence of anti-viral antibodies in the blood of patients who have successfully overcome the infection. Amazingly, in a recent newspaper article, a British professor of medicine wrongly referred to RT-qPCR as an “antigen” test. It is therefore not surprising that politicians, journalists and the general public fail to understand what tests are available, how they differ in methodology and the different uses they have in virus detection and patient surveillance. Clearly, one of the major questions then relates to the RT-qPCR-based testing procedures used to detect the presence of the SARS-CoV-2 genome in potentially infected individuals.

Other contentious topics are related to the number of assays that can be carried out, the time it should take to complete tests and report the results, as well as concerns associated with reagent shortages and how these issues initially prevented widespread and efficient testing of the population in some countries. Clearly, there is a lot of confusion about a number of issues:

The intention of this article is to provide basic information to the non-specialist about RT-qPCR and resolve some misconceptions about its application in the detection of SARS-CoV-2 RNA.

## 2. What Is the RT-qPCR Test?

**Figure 1:**
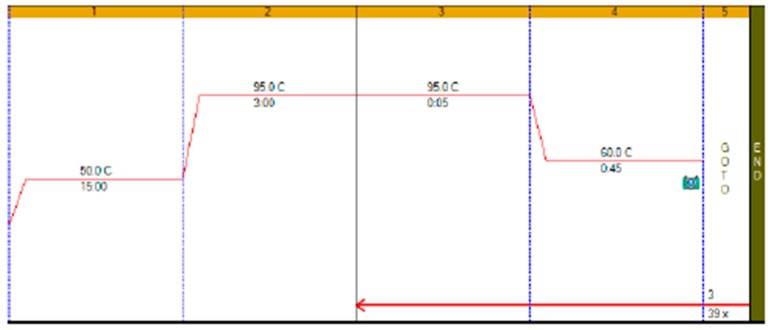
Thermal profile of a typical RT-qPCR test run on a BioRad CFX qPCR instrument. Here, the RT step is carried out at 50 °C for 15 min, followed by a 3-min RT deactivation and *Taq* polymerase activation step. The RT is followed by the PCR phase, which consists of a 5 s denaturation step, during which the DNA strands separate into single strands, and a 45 s 60 °C annealing/polymerisation incubation step, during which the amplification primers (and detection probes) hybridise to the single-stranded DNA templates and allow the polymerase to replicate the template, creating double-stranded DNA. During successful polymerisation, the probe is displaced and hydrolysed, separating fluorophore and quencher and releasing fluorescence. This process is repeated, usually around 40 times (40 cycles). A typical RT-qPCR run, as exemplified here, is completed in around 1 h 27 min. As this is a RT-qPCR run, quantification is achieved by measuring the intensity of fluorescence signals at the end of each cycle to deduce the amount of PCR product generated.

The polymerase chain reaction (PCR) is a highly sensitive and specific method for the amplification and detection of deoxyribonucleic acid (DNA) [1]. Its conceptual simplicity has made it the most widely used technique in molecular biology and it can, in theory, detect as little as a single fragment of DNA. Hence, it is widely used as a diagnostic test for a huge range of bacterial, fungal, viral and parasite pathogens. However, the genome of coronaviruses consists of ribonucleic acid (RNA) rather than DNA. Whilst RNA is similar to DNA, it is sufficiently different that *Taq* polymerase, the standard enzyme used for DNA amplification, replicates it only very inefficiently. Consequently, RNA is detected by a variant of the PCR test, termed reverse transcription (RT)-PCR [2].This encompasses a two-step method, typically comprising two enzymes; the first step uses a RNA-dependent DNA polymerase, also known as a reverse transcriptase, to copy RNA into DNA (cDNA), the second step then switches to the use of *Taq* polymerase, which amplifies the cDNA as in a standard PCR test (Figure 1).

**Figure 2:**
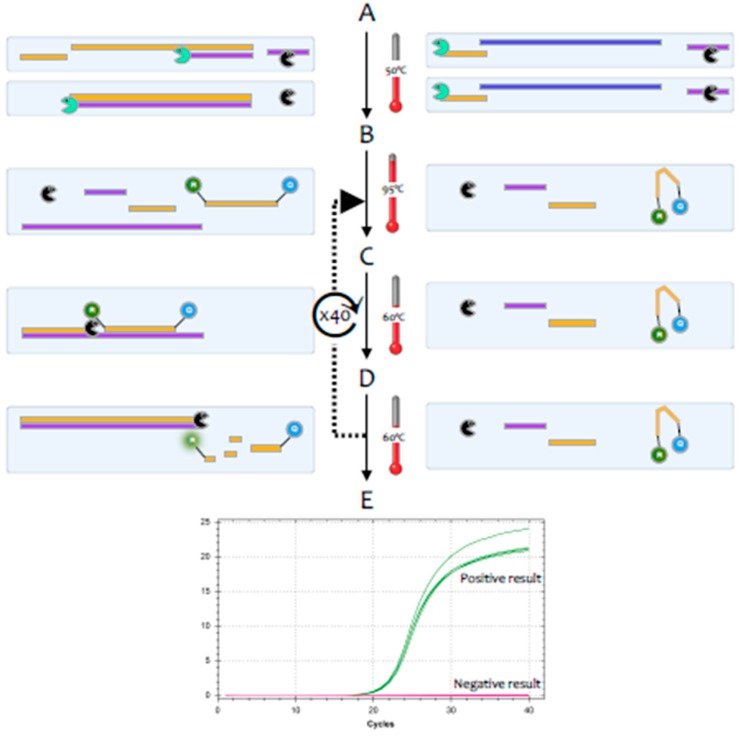
Signal generation during a RT-qPCR test. Test reagents include a buffer, both enzymes, target-specific DNA primers, and a target-specific DNA probe that is labelled at one end with a fluorescent label and at the other with a quencher. Samples on the left and right contain the same primers and probe, but the one on the left harbours target RNA, whereas the one on the right does not. **A.** RT: Samples are incubated at around 50 °C, which results in the RT transcribing target-specific cDNA from one of the strand-specific primers on the left, with no reverse transcription on the right. **B.** Denaturation: Samples are heated to 95 °C, which denatures the RNA but leaves the cDNA intact. **C.** Annealing: the temperature is lowered to around 60 °C, with the actual temperature assay-dependent. This allows both the target-specific primers and probe to bind to their respective targets on the left, whereas primers and probe remain unbound on the right. **D.** Polymerisation: this step may be combined with the annealing step. On the left, the polymerase extends DNA synthesis, initially from one primer only, but after the first cycle from both, and displaces and hydrolyses any bound probe. This separates fluorophore and quencher and results in the emission of light if the fluorophore is excited at the appropriate wavelength. On the right, none of this occurs, and no light is emitted. This first cycle is followed by a further, user-defined number of cycles, indicated by the stippled arrow leading back to step B. **E.** Amplification plots obtained for each sample track the increasing emission of light characteristic of a positive result from the sample on the left (green plot), whereas the sample with no amplifiable target on the right records no light emission and a negative result (red plot).

For diagnostic purposes, it is most convenient to carry out the RT and the PCR reactions in a single test tube; for research use, the two steps are often carried out in separate tubes. There is an alternative approach that uses *Tth* polymerase, a thermostable enzyme that can replicate both RNA and DNA to carry out both the RT and PCR reactions [3], but this method tends to be less sensitive. Most diagnostic tests use a particular version of the RT-PCR test, termed fluorescence-based quantitative RT-PCR (RT-qPCR) [4] (Figure 2).

One of the valuable advantages of RT-qPCR is the ease with which RNA in general, and viral load specifically, can be quantified, if adequate assay parameters are established and appropriate controls are included [5]. The quantification cycle (Cq) is at the heart of accurate and reproducible quantification using RT-qPCR. Fluorescence values are recorded during every cycle and represent the amount of product amplified up to that point in the amplification reaction. The more template present at the beginning of the reaction, the fewer cycles it takes to reach a point at which the fluorescent signal is first recorded as statistically significant above background. This point is defined as the Cq and will always occur during the exponential phase of amplification. Therefore, quantification is not affected by any reaction components becoming limited in the plateau phase [2]. However, it is important not to rely solely on the Cq when reporting results, as Cq values are subject to inherent inter-run variation [6] and should not be used without appropriate calibration standards [5]. One obvious way to achieve reliable quantification is to include an RNA molecule of known copy number as a spike with the RNA following extraction. This would allow both a measure of quality control, as any deviation from the expected Cq would suggest some inhibition of the reaction, as well as determination of viral copy number relative to that spike. This makes it possible to report not just a qualitative infected/not infected answer but to aim to include an assessment of viral load to measure disease progression, for example.

Ultimately, the reliability of RT-qPCR results is dependent on the standardisation of measurements [7], especially so when used as a diagnostic tool. It is clear that the need to be able to compare results from a wide range of tests, instruments, different laboratories and different countries makes it essential to develop reference materials, which would ultimately be certified, to allow for harmonisation of data.

Recent years have seen the advance of digital PCR as a complementary approach for measuring nucleic acids that can be highly reproducible when performed at different times and when different primer sets are targeting the same molecule, as is the case with SARS-CoV-2 [8]. Whilst cost of instrumentation, throughput, infrastructure requirements and penetration of RT-dPCR cannot compare to RT-qPCR, it is likely that this method is useful as a confirmatory method for suspected cases of SARS-CoV-2 infection [9,10], especially when detecting very low viral loads.

## 3. What Reagents Are Required to Carry it Out?

A complete RT-qPCR assay requires few components, each of which are available in abundance. RNA extraction reagents use a number of standard chemicals, including guanidinium isothiocyanate and Triton surfactants, and there is no shortage of RTs, *Taq* polymerases, primers and probes or components for the RT-qPCR buffer. This makes the UK government’s claim of a shortage of chemical reagents delaying adequate testing rather surprising.

Furthermore, even if there were a temporary interruption of supplies, most laboratories are well-stocked and, as with PCR protocols, significant reductions in the amounts of reagents used per test are easily achieved. Many standard reactions are carried out in 25-µL volumes, but it is perfectly feasible to achieve the same sensitivity and accuracy in reaction volumes as little as 5 µL. As Table 1 shows, this leads to a significant decrease in the amount of enzyme and buffer master mix required, with the additional benefit of significant cost reduction per test. A quantity of 25 mL of enzyme mix, sufficient for 10,000 tests, has a list price of around £925 in the UK (PCRBio OneStep RT-qPCR master mix).

## 4. How Long Does It Take to Generate Results?

**Figure 3:**
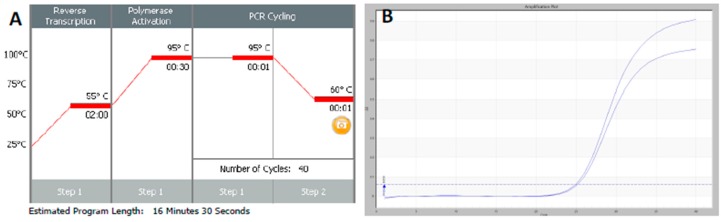
Amplification of SARS-CoV-2 using a fluorescence-based RT-qPCR assay. **A.** Thermal profile of a typical fast RT-qPCR test. The RT step can be carried out at a range of temperatures, here Superscript IV at 55 °C, for different lengths of time, here 2 min. A polymerase activation step deactivates the RT and activates *Taq* polymerase, here SensiFast (Bioline), with the time dependent on which polymerase is being used. PCR cycling steps in this example are 40 cycles at 95 °C (denaturation) and 1 s at 60 °C (annealing/polymerisation), allowing the RT-qPCR run to be completed in around 17 min. As this is a RT-qPCR run, at the end of each cycle fluorescence intensity is read to establish the amount of PCR product generated. B. Amplification plot generated using the above protocol, with the assay run as a duplicate RT-qPCR using OneStep (PCRBio) kit. The primer and probe sequences were (5′-3′) F: GGATCAAGAATCCTTTGGTGG; R: GTCACAAAATCCTTTAGGATTTGGA; Probe: FAM-. CATCGTGTTGTCTGTACTGCCGTTGCC-BHQ.

RT-qPCR protocols have not changed very much over the past twenty years, with few diagnostic kits making use of the enormous advances that have taken place with regards to increases to instrument ramp rates or enzyme polymerisation speed and processivity. Consequently, most RT-qPCR assays still take between 45 to 90 min to complete, as shown in Figure 1. It is possible to reduce this to below 20 min by a simple change of protocol [11]. This involves designing assays that generate PCR products of 60–80 bp, using fast RNA- and DNA-dependent DNA polymerases such as Superscript IV and KAPA *Taq* polymerase, respectively, and selecting instruments capable of rapid cycling, for example Eco from PCRMax. This allows the RT step to be limited to 2 min or less, with the denaturation and annealing/polymerisation steps limited to 1 s each (Figure 3). As a result, a test can be completed in well below 20 min, with most time taken up by the instrument reading fluorescence level after each cycle. If real-time results are not required, this run could be completed in less than 10 min. Even faster run times can be achieved with other microfluidics-based instruments and experimental sub-20 s cycling times for qPCR assays (“extreme PCR”) have been demonstrated [12], pointing towards future virtually instantaneous PCR results.

However, it is important to remember that validation and verification of laboratory workflows, and ensuring their compliance with international standards, are essential components of safe and reliable molecular diagnostic testing. They help ensure comparability and minimise the risk of false reporting. However, the speed and scale of the COVID-19 epidemic has resulted in a relaxation of the strict rules governing procedures carried out in accredited and other clinical diagnostic laboratories, with the FDA opening an emergency use authorisation process for regulated assay development in the current emergency. Clearly, a top consideration for using any new or modified tests is that they report accurately and sensitively, reducing the risk of recording false positive or false negative results. There is an urgent need to generate minimum validation and reporting standards, a process that is underway by the Coronavirus Standards Working Group, launched by the Joint Initiative for Metrology in Biology (https://jimb.stanford.edu/covid-19-standards).

## 5. What Is the Expected or Available Throughput?

Whilst most qPCR instruments are able to simultaneously process 96 reactions, there are diagnostic instruments capable of analysing 384 and even 1536 samples through a single test. Consequently, even with conventional slow protocol times of around 1.5 h, a single one of these instruments can, at least in theory, deal with many thousands of samples daily, respectively, with a fairly modest halving of the run time doubling that throughput (Table 2). Since run times of less than 20 min are easily achieved on some instruments, this could be doubled to more than 4000, 18,000 and 70,000 per instrument. Even 48-well instruments, which are not generally regarded as capable of high throughput, can perform an unexpectedly high number of tests if protocols are adjusted for speed, as discussed above. As shown in Table 2, a 20-min RT-qPCR protocol similar to the one shown in Figure 3 on a 48-well instrument, results in a theoretical test capacity of more than 3000 tests per day for that one instrument.

Since there are thousands of qPCR instruments available in all advanced economies, there is adequate facility for RT-qPCR testing of the clinical samples reported that can be run every day, with the UK government target of 100,000 tests per day in theory achievable on a single instrument. Of course, in practice, each instrument will be used for far fewer tests, but these calculations show that there is no shortage of capacity to accomplish consistent, high-throughput genomic testing in patients, healthcare workers and, indeed, the general population. Obviously, there are a large number of different systems in different states of repair, and not all instruments or locations will be suitable for diagnostic testing. However, with many hospital pathology departments having suitable instruments, infrastructure and expertise and many research institutes, universities and pharma companies having well-stocked and expert core facilities, in addition to equipment being available in individual laboratories, there is the potential to significantly increase testing capacity whilst maintaining high testing and reporting standards. Certainly, the inclusion of known negative and positive control samples with each test run is an essential quality control parameter.

The bottleneck is likely to be the RNA extraction process, but again there are hundreds, if not thousands of robots available that can extract 96 samples at a time in around one hour. Given a 24 h schedule, each robot could prepare RNA from more than 2300 samples every day. Moreover, public health laboratories, academic core facilities and many large commercial and major research laboratories have more than one of those robots. The UK alone has more than 600 containment level 3 laboratories, which should allow centralised large-scale RNA preparation under containment level 3 conditions. The non-infectious RNA could then be forwarded to multiple testing sites.

It is worth mentioning isothermal amplification in general and LAMP (loop-mediated isothermal amplification) [13] in particular here, as this method is less sensitive to inhibitors and so can be used on more crude samples with minimal extraction of nucleic acid. LAMP and other isothermal systems are not dependent on different temperatures and so are suitable for application at the point of care. At least one LAMP-based assay targeting SARS-CoV-2 has already been published [14], and others are being tested (for example a system developed by TATAA Biocenter and Danish Technical University) so should be able to add to the testing potential without impinging on the extraction capacity.

## 6. What Other Considerations Are There?

Clinical laboratory testing has progressed a long way since external quality assessments (EQA) first identified problems in comparing results between different clinical laboratories [15]. Today there are standards developed by the International Organization for Standardization, including the 20166 series, which are specifically designed for molecular in vitro diagnostic examinations, as well as standards such as ISO 20395:2019, which lists the requirements for evaluating the performance of quantification methods for nucleic acid target sequences with regards to qPCR and dPCR. The objective of these standards is to reduce the impact of external factors on the results of examinations, thus ensuring that patients obtain diagnoses that are as objective as possible.

The preanalytical stage of molecular testing, which involves sample collection, handling and storage and the potential for sample contamination [16] as well as issues around nucleic acid extraction [17] is a major source of errors in diagnostic laboratory testing. Samples for PCR testing for COVID-19 are usually taken from inside the nose, the mouth or the back of the throat, and careful sampling and nucleic acid preparation are important pre-test considerations. It is clearly necessary to maximise the likelihood of successfully collecting virus by meticulous sampling. Furthermore, since RT-qPCR is inhibited by many substances present in human samples [18], it is important to use nucleic acid extraction methods that remove these inhibitors. Obviously, both criteria are especially crucial to avoid false negative results. It has also become apparent that some commercial test kits, and probes and primers in particular, have been contaminated with SARS-CoV-2 sequences, with important implications for false positive detection.

The efficiency of a RT-qPCR test is of crucial importance. There is a wide choice of enzymes that can carry out the two steps, and they all differ in their properties. The RNA conversion step is notoriously variable, and some RT-PCR enzyme combinations are more efficient than others [19]. Since the PCR step amplifies DNA in an exponential manner, small differences in efficiency can result in large differences in test sensitivity. Again, this is most important during the very early stages of infection, when a swab may collect very few virus particles.

The reliability of the test is also dependent on the reagents used to allow the enzyme to amplify and detect its target. These are the SARS-CoV-2-specific primers and probe, which must be 100% specific for the virus and so amplify only viral sequences and report the increasing amount of PCR amplicon being synthesised. There are several viral genomic regions targeted by RT-qPCR assays, and multiple primer designs targeting the same genes. These include the *RdRP* gene (RNA-dependent RNA polymerase gene), the *E* gene (envelope protein gene), and the *N* gene (nucleocapsid protein gene). Importantly, it should be noted that two different sets of primers can both be 100% specific for their target but show different amplification efficiencies and so result in different sensitivities of the assays [20]. This has been observed for SARS-CoV-2 assays, where differences in the performance of the various tests have been reported [21,22] which may be due to differences in priming efficiency or protocols used, or may be associated with viral RNA secondary structure or stability. Primers are arguably the single most critical component of a reliable RT- qPCR assay, as their properties control the exquisite specificity and sensitivity that make this method uniquely powerful [19]. This is especially so for the single-tube RT-qPCR assays generally used to detect SARS-CoV-2, as the viral RNA has extensive secondary structure, which has a substantial impact on the efficiency of reverse transcription and hence the sensitivity and reliability of the whole assay [5]. Consequently, poor design combined with failure to optimise reaction conditions is likely to account for false negative results, with variable RNA sequences within the *ORF1ab* gene and *N* genes one possible cause for negative or low sensitivity results [23].

## 7. Conclusions

RT-qPCR testing programmes for SARS-CoV-2 are wholly inadequate, poorly organised and surrounded by confusion and misinformation. Comprehensive testing is not hindered by availability of suitable assays, reagents, equipment or testing capacity. It is delays in the bureaucratic validation and approval process and lack of involvement of the wider research and commercial service provider community by public health laboratories that are at the heart of the testing conundrum. An immediate solution would be to centralise RNA extraction procedures in containment level 3 facilities, but devolve testing of non-infectious RNA to nation-wide laboratories, where each country has a large pool of experienced staff willing to carry out the actual testing. For future pandemics, there is an obvious need for public health organisations such as the US CDC and UK PHE to learn some significant lessons and establish protocols for emergency testing systems that can be rapidly adopted in emergency situations. For now, the Coronavirus standards working group (https://jimb.stanford.edu/covid-19-standards), led by the Joint Initiative for Metrology in Biology, is developing a set of guidelines to ensure the availability of common, appropriate standards, controls, validation tests and protocols that are essential for the accuracy of test results. They also aim to develop an annotated inventory of resources, identify gaps, and conduct international collaborative experiments to evaluate and establish comparability of the diverse control materials and test methods available. The group will institute an open information repository of validation methods and data. This should lead to collaboratively produced reference materials and samples as well as reference measurement methods to secure an enduring and reliable COVID testing enterprise.

## Figures and Tables

**Table 1:** Reduction in the amounts of enzyme mix required for RT-qPCR tests by reducing assay volumes.

Volume of RT-qPCR	Enzyme Mix/Test	Enzyme Mix/10,000 Tests
(µL)	(µL)	(mL)
25	12.5	125
10	5.0	50
5	2.5	25

Grey colour: a contrast.

**Table 2:** Maximum capacity of a single qPCR instrument, assuming it is run for 24 h a day. A. A typical diagnostic instrument such as the Roche Lightcycler. B. An extremely fast research qPCR instrument with 48 wells (PCRMax Eco). This instrument stands out as it is one of the fastest qPCR systems available and typical research qPCR systems do not perform like this. More typical research qPCR systems include the Biorad CFX systems, Applied Biosystems StepOnePlus/QuantStudios, etc. All of these are capable of running standard and fast protocols but do not have the higher ramp rates of the Eco to perform such very rapid PCR.

**PCR Instrument**	**Reactions/Run**	**Daily Throughput**	**Daily Throughput**
e.g., Roche Lightcycler		1.5 h/run	20 min/run
Low throughput	96	1536	6912
Medium throughput	384	6144	27,648
High throughput	1536	24,576	110,592
**Rapid 48 Well Instrument**	**Reactions/Run**	**Daily Throughput**	**Daily Throughput**
e.g., PCRMax Eco		45 min/run	20 min/run
Throughput	48	1536	3456

Grey colour: a contrast.
